# Micro-CT protocols for scanning and 3D analysis of *Hexaplextrunculus* during its different life stages

**DOI:** 10.3897/BDJ.9.e71542

**Published:** 2021-09-15

**Authors:** Eva Chatzinikolaou, Kleoniki Keklikoglou

**Affiliations:** 1 Hellenic Centre for Marine Research (HCMR), Institute of Marine Biology, Biotechnology and Aquaculture (IMBBC), Heraklion, Crete, Greece Hellenic Centre for Marine Research (HCMR), Institute of Marine Biology, Biotechnology and Aquaculture (IMBBC) Heraklion, Crete Greece; 2 Biology Department, University of Crete, Heraklion, Crete, Greece Biology Department, University of Crete Heraklion, Crete Greece

**Keywords:** micro-CT, gastropod, shell, statolith, morphology, methodology, scanning, *
Hexaplex trunculus
*

## Abstract

Micro-computed tomography (micro-CT) is a high-resolution 3D-imaging technique which is now increasingly applied in biological studies focusing on taxonomy and functional morphology. The creation of virtual representations of specimens can increase availability of otherwise underexploited and inaccessible samples. The 3D model dataset can be also further processed through volume rendering and morphometric analysis. The success of micro-CT as a visualisation technique depends on several methodological manipulations, including the use of contrast enhancing staining agents, filters, scanning mediums, containers, exposure time and frame averaging. The aim of this study was to standardise a series of micro-CT scanning and 3D analysis protocols for a marine gastropod species, *Hexaplextrunculus*. The analytical protocols have followed all the developmental stages of this gastropod, from egg capsules and embryos to juveniles and adults.

## Introduction

Micro-computed tomography (micro-CT) is a high-resolution technique, based on X-ray 3D-imaging which allows non-destructive studies of external and internal structures of a specimen. Micro-CT has been more extensively used in geological and paleontological studies, but it is now often used as a tool in biological studies focusing on taxonomy, evolution and functional morphology (Faulwetter et al. 2013). The creation of virtual representations of specimens and especially of natural history museum specimens, could offer a widely available and fast access to valuable and sensitive specimens, which otherwise would be underexploited and inaccessible (Blagoderov et al. 2012).

A specimen is placed between an X-ray source and an X-ray detector in the micro-CT scanner, resulting in the acquisition of a large set of 2D projections (2D images) of the object around a rotation axis which can be then reconstructed into cross-section images. A full virtual representation of the specimen is created as a 3D model, which can be interactively manipulated on screen (rotation, zoom, virtual dissection and isolation of specific features of interest) (Keklikoglou et al. 2019). The micro-CT dataset can then be processed for 3D visualisation through volume rendering and a series of 3D measurements (e.g. size, volume density, porosity, thickness) and comparative morphometric analyses can be performed on the derived 3D models.

The success of micro-CT as a visualisation technique depends initially on achieving a sufficient contrast difference between the specimen and its surrounding medium, as well as between the different specimen tissues (e.g. hard structures versus soft tissues) (Keklikoglou et al. 2019). Contrast enhancement agents (i.e. staining), as well as specialised aluminium or copper filters, can be used to improve the quality and clarity of the scanning results. The selection of an appropriate contrast agent is a combination of several factors, such as the tissue type, the fixation and storage medium, the penetration rate and the acidity of the contrast agent (Metscher 2009). For example, contrast agents with a high atomic number result in an increased absorption of X-rays, although staining agents with low penetration rates can be proven to be more effective when used in smaller specimens (Pauwels et al. 2013). Acidic agents might cause dissolution of calcified tissues, such as bones or shells, while agents dissolved in ethanol would require a gradual dehydration of the specimen stored in water in order to avoid shrinkage (Pauwels et al. 2013). Scanning quality of specimens, including a combination of both hard and soft tissues, can be improved when a filter is used. However, the use of filters might reduce density values and increase noise level and, thus, in such cases, a reduction in voltage can improve tissue contrast and image quality (Meganck et al. 2009).

The appropriate selection of several parameters, including voltage and current applied, scanning medium and container, use of filters and/or staining agents, exposure time, magnification, degrees of specimen rotation and rotation step, frame averaging etc., are important for the achievement of the optimum imaging result. Therefore, the aim of this study was to establish and standardise a series of micro-CT scanning and 3D analysis protocols for a marine gastropod species, *Hexaplextrunculus* (Linnaeus, 1758). This is a common and widely distributed gastropod, well-adjusted to varying physical environmental factors and has an important economic value in several countries.

The analytical protocols have followed all the developmental stages of this gastropod, from egg capsules and embryos to juveniles and adults.

## Implementation and Methodology

### Scanning equipment

All scans were performed with a SkyScan 1172 micro-tomograph (Bruker, Kontich, Belgium) at the Hellenic Center for Marine Research (HCMR), Institute of Marine Biology, Biotechnology and Aquaculture (IMBBC), Heraklion, Crete. The scanner uses a tungsten source and is equipped with an 11MP CCD camera (4000 x 2672 pixels), which can reach a maximal resolution of < 0.8 m m/pixel.

### Scanning protocols for the shells of adult Hexaplextrunculus

Adult *Hexaplextrunculus* were anaesthetised with 7% magnesium chloride (MgCl_2_) and stored in the freezer (-20°C). Samples (shell including tissue) were scanned without any staining, inside a custom-made felisol sample holder without any scanning medium. Felisol has a low X-ray absorption and, therefore, did not affect measurements under the selected scanning parameters. Specimens were scanned at a voltage of 100 kV and a current of 100 μA using a combined aluminium and copper filter. Images were acquired at a pixel size of 13.79 μm with a camera binning of 2 × 2. Exposure time was 2480 ms and scans were performed for a half rotation of 180° (rotation step 0.60°) in order to minimise the scanning duration. Initial tests had shown that there was no significant loss of information or increase of artefacts compared to a full 360° rotation. Scanning was performed without frame averaging. Scanning duration time was ~ 5 hours and 20 minutes.

**Figure 1.**
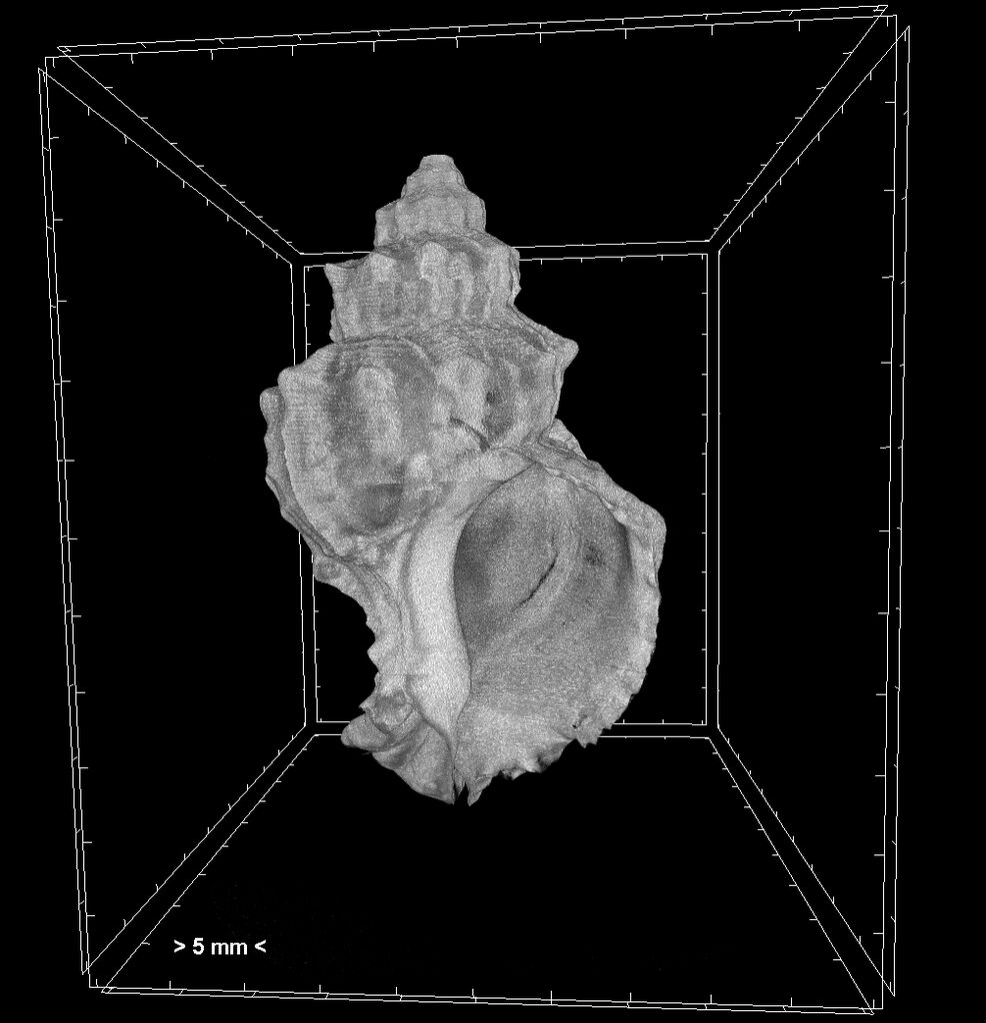
Volume rendering of an adult *Hexaplextrunculus*.

Projection images were reconstructed into cross sections using the SkyScan’s NRecon software (Bruker, Kontich, Belgium) which employs a modified Feldkamp’s back-projection algorithm. All scans were reconstructed using the same range of attenuation coefficients (0 - 0.13) in order to obtain comparable results between samples. The reconstructed images were stored as 16-bit TIFF images. Volume renderings of each specimen were created using the CTVox software (Bruker, Kontich, Belgium) in order to display the reconstructed images as a 3D object (Fig. 1).

### 3D analysis and data generation

The cross section images were loaded into the software CT Analyser v.1.18.4.0 (CTAn, Bruker, Kontich, Belgium). The mean greyscale values of the total shell were calculated using the binary threshold module which allows for comparable measurements of the relative density of the calcified tissues (i.e. shell). For the present analyses, no absolute density values (e.g. Hounsfield units) were required, since comparability between the scans by the calculation of relative densities was considered to be sufficient. Relative grey scale density in the present study is used as a proxy for "micro-density" (i.e. density of the shell material including calcium carbonate (CaCO_3_) and intraskeletal organic matrix) and not to bulk density which includes porosity. The range of the greyscale histogram was 30 - 255 for all shell specimens of adult *Hexaplextrunculus*.

**Figure 2.**
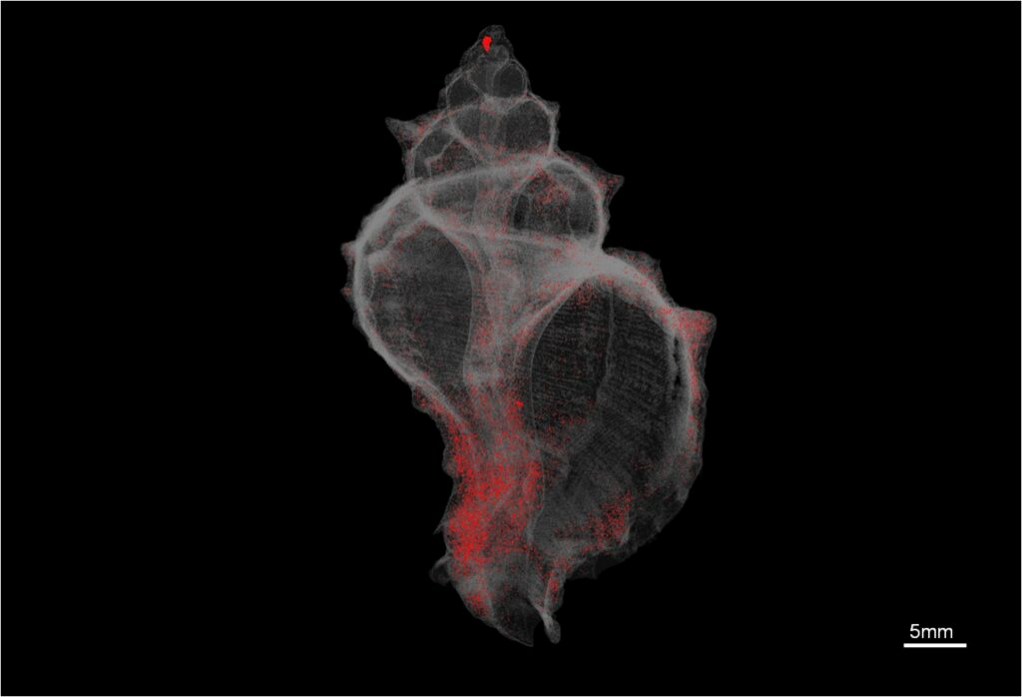
3D model of the closed pores of the adult *Hexaplextrunculus*, indicated in red.

**Figure 3.**
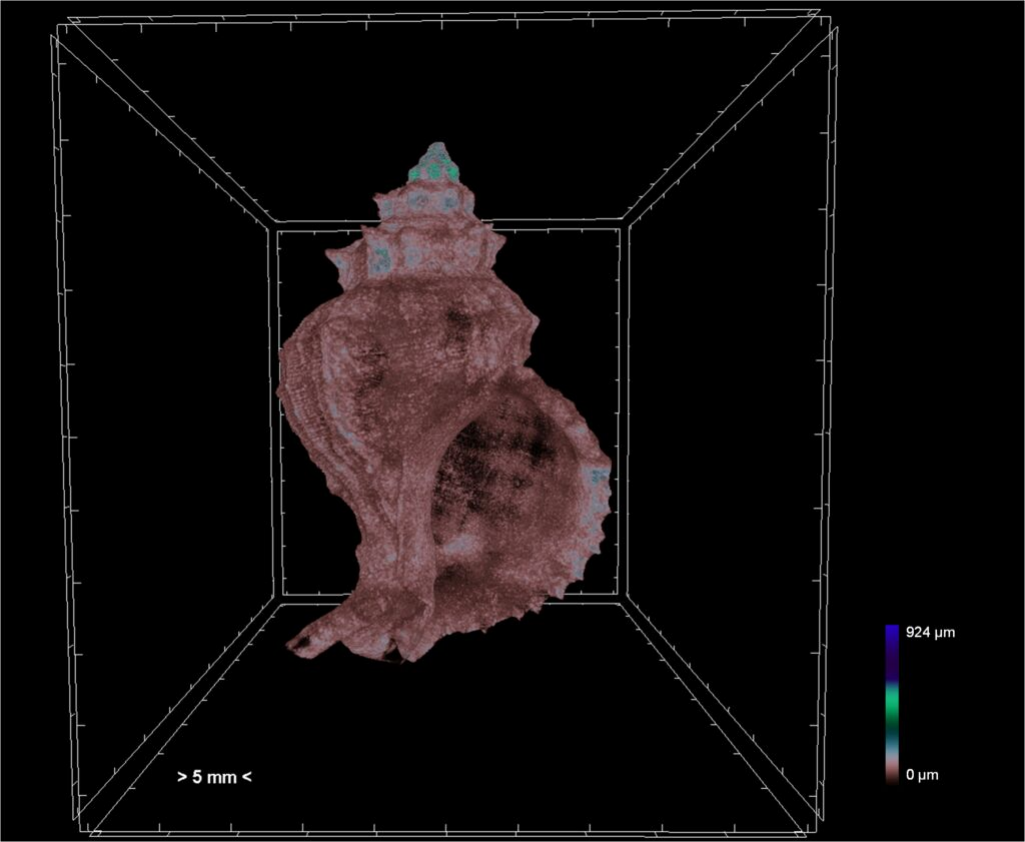
Colour-coded image of adult *Hexaplextrunculus* of structure thickness. Warmer colours (red) indicate thinner structures and cooler colours (blue) indicate thicker structures.

Furthermore, the 3D analysis was performed with the custom processing plugin of the CTAn software, using the volume of interest (VOI) in each specimen in order to calculate the porosity and the structure thickness. Porosity was calculated as the percentage of the closed porosity of the shell (i.e. total volume of enclosed pores of each specimen as a percentage of the total shell volume) (Fig. 2). Structure thickness of the shell (Fig. 3) was calculated as the average of the diameters of the largest spheres which can be fitted into each point of the shell structure ("sphere-fitting" method) (Hildebrand and Rüegsegger 1997).

3D geometric morphometric methods were applied on the surface model of each specimen, which was created using the CTAn software. Subsequently, these surface models were loaded into the Landmark Editor software in order to add landmarks as single points and curves in specific shell areas, which will be used for the morphological shape comparisons. Comparable measurements of shape were performed by reproducing the same landmark protocol in all shell specimens. All data points were loaded into the MorphoJ software where models were adjusted in a "procrustes fit" in order to conduct a Procrustes Analyses.

The protocols for adult *Hexaplextrunculus* have been published under https://www.protocols.io (dx.doi.org/10.17504/protocols.io.bxwqppdw).

### Scanning protocols for the egg capsules of Hexaplextrunculus

Egg capsules of *Hexaplextrunculus* were fixed in 5% formaldehyde, buffered with seawater for 4 - 5 days. The capsules were washed with distilled water and then were dehydrated with ethanol in gradually increasing concentrations (20%, 50%, 70%, 96%). Finally, the capsules were stained with 1% iodine in 96% ethanol for 7 days. Specimens were placed inside a plastic Falkon tube and scanning was performed in 96% ethanol as a scanning medium. Specimens were scanned at a voltage of 80 kV and a current of 124 μA using an aluminium filter. Images were acquired at a pixel size of 13.79 μm with a camera binning of 2 x 2. Exposure time was 1435 ms and scans were performed for a full rotation of 360° (rotation step 0.40) with a frame averaging set at 3. Scanning duration time was ~ 3 hours and 38 minutes.

**Figure 4.**
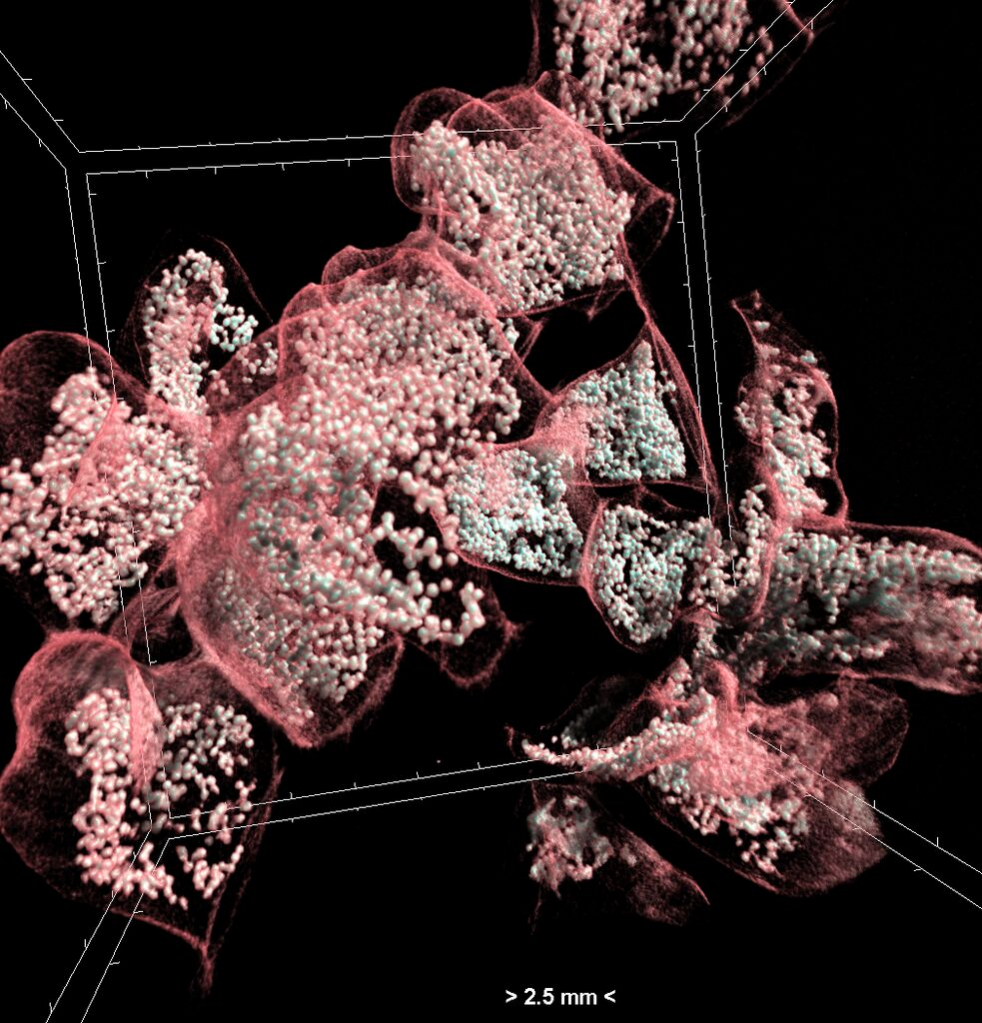
Volume rendering of egg capsules of *Hexaplextrunculus*. The capsule walls and the eggs included are visible.

Projection images were reconstructed into cross sections using SkyScan’s NRecon software (Bruker, Kontich, Belgium) which employs a modified Feldkamp’s back-projection algorithm. All scans were reconstructed using the same range of attenuation coefficients (0 - 0.064) in order to obtain comparable results. The reconstructed images were stored as 16-bit TIFF images. Volume renderings of the egg capsules were created using the CTVox software (Bruker, Kontich, Belgium) in order to display the reconstructed images as a 3D object (Fig. 4).

The scanning protocols for the egg capsules of *Hexaplextrunculus* have been published under https://www.protocols.io (dx.doi.org/10.17504/protocols.io.bxw5ppg6).

### Scanning protocols for the embryos and juveniles of Hexaplextrunculus

Embryos of *Hexaplextrunculus* were removed from their egg capsules, anaesthetised with 7% MgCl_2_ and scanned without any staining, inside a plastic white pipette tip and without any scanning medium. Specimens were scanned at a voltage of 59 kV and a current of 167 μA without using any filters. Images were acquired at a pixel size of 2 μm with a camera binning of 1 × 1. Similarly, juveniles of different ages (2-8 months old) were scanned using the same parameters and settings. Exposure time was 325 ms for embryos and 316 ms for juveniles. Scans were performed for a full rotation of 360° (rotation step 0.20) with a frame averaging set at 3 for embryos and for a 180° rotation (rotation step 0.25) with a frame averaging set at 5 for juveniles, respectively. Scanning duration time was ~ 2 hours and 23 minutes.

**Figure 5.**
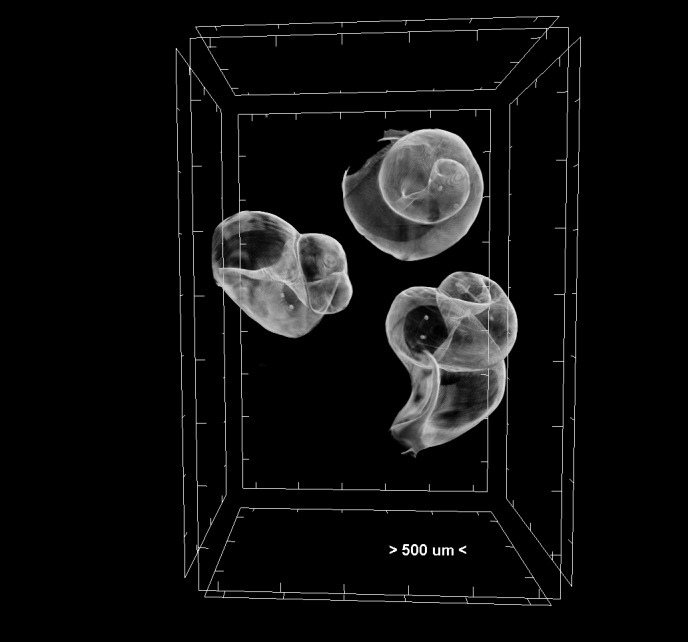
Volume rendering of gastropod embryos of *Hexaplextrunculus*. The pair of statoliths can be seen as two white dots inside the shell of each specimen.

**Figure 6.**
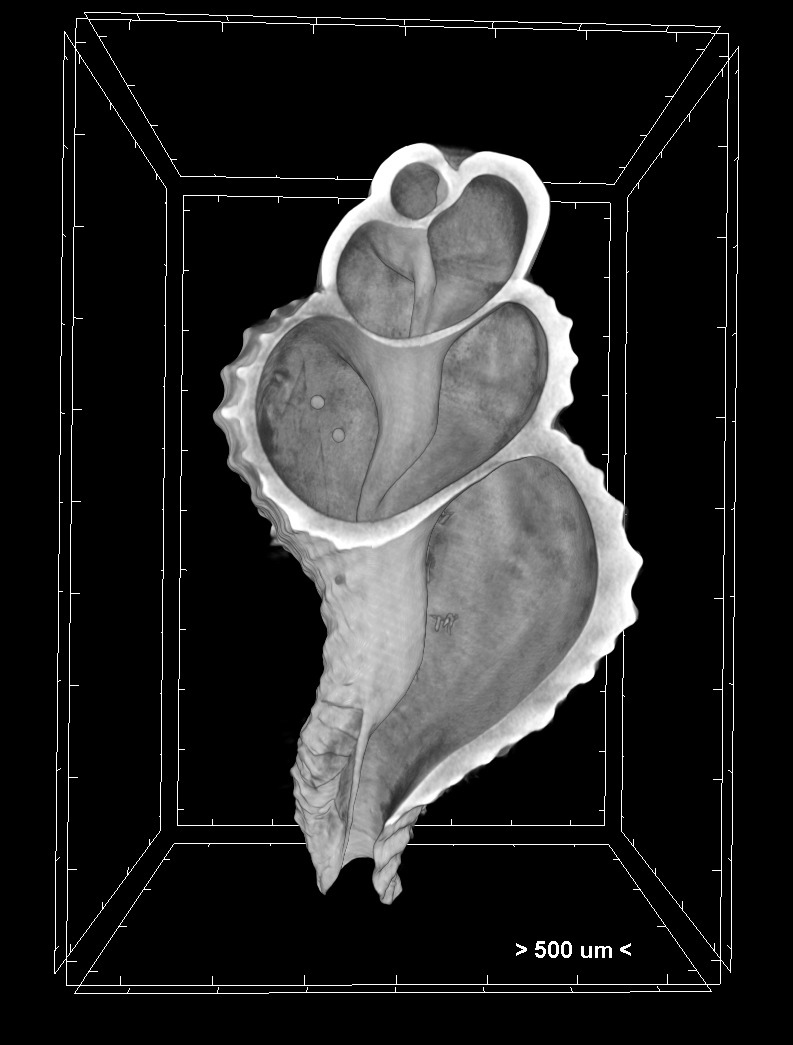
Volume rendering of a 6.5 months old juvenile *Hexaplextrunculus*. The pair of statoliths can be clearly seen on the left side of the specimen.

Projection images were reconstructed into cross sections using SkyScan’s NRecon software (Bruker, Kontich, Belgium) which employs a modified Feldkamp’s back-projection algorithm. All scans were reconstructed using the same range of attenuation coefficients (0 - 1.127 for embryos and 0 - 1 for juveniles) in order to obtain comparable results. The reconstructed images were stored as 16-bit TIFF images. Volume renderings of the embryos and juveniles were created using the CTVox software (Bruker, Kontich, Belgium) in order to display the reconstructed images as a 3D object (Fig. 5 for embryos and Fig. 6 for juveniles).

The scanning protocols for the embryos and juveniles of *Hexaplextrunculus* have been published under https://www.protocols.io (dx.doi.org/10.17504/protocols.io.bxw4ppgw).

## Conclusions

The use of micro-CT for studying the structural and morphological properties of the gastropod shell has been proven as a valuable tool, which can be successfully applied throughout the different developmental stages of this gastropod species. In addition, the micro-CT generated volume renderings revealed the internal calcareous statolith structures of embryos and egg capsules of *Hexaplextrunculus*, since they have a similar X-ray absorption with their shells. However, statoliths were not visible in the scans of the adult specimens due to the higher density of their shells, thus requiring manual dissection in order to be examined. The accurate visualisation of 3D structures can offer a significant insight into comparative, functional and developmental studies of animal morphology (Metscher 2009). Previous studies have also used this method for analysing shells of the marine gastropods *Nassariusnitidus* and *Columbellarustica*, where specimens were collected from different experimental designs investigating the effect of ocean warming and acidification on the shell density, porosity and thickness (Chatzinikolaou et al. 2017, Chatzinikolaou et al. 2021). Golding et al. (2009) investigated the odontophoral cartilages supporting the movement of the radula during feeding in Caenogastropoda in order to investigate phylogeny and for understanding the biomechanical operation of the buccal mass and the adaptation to trophic specialisation. Taxonomy and phylogeny of terrestrial and fossil snails have been also supported by micro-CT studies (Afriat et al. 2021, Yu et al. 2021, Yu et al. 2019). Micro-CT morphological studies on small-sized zoological specimens is a well developing and promising field with many applications through different scientific disciplines (Jochum et al. 2019, Jochum et al. 2015, Stoev et al. 2013).

The present manuscript presented some specific guidelines regarding scanning of the marine gastropod *Hexaplextrunculus* at different developmental stages. The appropriate protocol depends on the species and the tissue or organ targeted, as well as the selected experimental design and the hypothesis tested in each case study. However, some general considerations to be taken in mind can be derived from the present study. Shelled organisms do not need any type of sample preparation for visualisation of their hard structures, while egg capsules need to be stained prior micro-CT scanning. Internal structures (e.g. statoliths) can be better studied in small-sized juveniles which have thinner shells. Investigation of internal soft tissue organs in thicker-shelled organisms will require removal of the body and discarding of the shell. The scanning parameters used (e.g. voltage, current, filter, camera binning, exposure time) are species- and size- specific. Larger gastropods have thicker shells with higher X-ray absorption and they will require the use of filters in order to avoid the presence of artifacts. These protocols could serve as a guide and a starting basis for other molluscan species. By sharing common methodologies, the establishment of a comprehensive protocol for the study of gastropod's morphology could be developed and comparative results can be derived throughout several studies.

## Hosting institution

Hellenic Centre for Marine Research - Institute of Marine Biology, Biotechnology and Aquaculture
